# *De novo* transcriptome characterization of Vitis vinifera cv. Corvina unveils varietal diversity

**DOI:** 10.1186/1471-2164-14-41

**Published:** 2013-01-18

**Authors:** Luca Venturini, Alberto Ferrarini, Sara Zenoni, Giovanni Battista Tornielli, Marianna Fasoli, Silvia Dal Santo, Andrea Minio, Genny Buson, Paola Tononi, Elisa Debora Zago, Gianpiero Zamperin, Diana Bellin, Mario Pezzotti, Massimo Delledonne

**Affiliations:** 1 Biotechnology Department, University of Verona, Strada Le Grazie 15, I-37134, Verona, Italy

**Keywords:** Transcriptomics, RNA-Seq, *de novo* assembly, Grape, Varietal diversity

## Abstract

**Background:**

Plants such as grapevine (Vitis spp.) display significant inter-cultivar genetic and phenotypic variation. The genetic components underlying phenotypic diversity in grapevine must be understood in order to disentangle genetic and environmental factors.

**Results:**

We have shown that cDNA sequencing by RNA-seq is a robust approach for the characterization of varietal diversity between a local grapevine cultivar (Corvina) and the PN40024 reference genome. We detected 15,161 known genes including 9463 with novel splice isoforms, and identified 2321 potentially novel protein-coding genes in non-annotated or unassembled regions of the reference genome. We also discovered 180 apparent private genes in the Corvina genome which were missing from the reference genome.

**Conclusions:**

The *de novo* assembly approach allowed a substantial amount of the Corvina transcriptome to be reconstructed, improving known gene annotations by robustly defining gene structures, annotating splice isoforms and detecting genes without annotations. The private genes we discovered are likely to be nonessential but could influence certain cultivar-specific characteristics. Therefore, the application of de novo transcriptome assembly should not be restricted to species lacking a reference genome because it can also improve existing reference genome annotations and identify novel, cultivar-specific genes.

## Background

Grapevine is the most cultivated fruit crop in the world, covering approximately 7.8 million hectares in 2011 and producing more than 67 million tons of berries (http://www.fao.org). The modern grapevine (Vitis vinifera sbs. sativa) was domesticated 8000 years ago in the Southern Caucasus region [1] from its wild ancestor V.vinifera sbs. sylvestris. The grapevine genome is highly polymorphic [2] and vegetative propagation is preferred over seed germination because the extensive heterozygosity results in erratic yields and produces offspring with diverse characteristics [3]. At least 14,000 grapevine varieties have been cataloged [4] but breeding is restricted to a relatively small number of cultivars, such as Pinot and Traminer [5]. However, the global demand for high-quality wines is increasing, awakening interest in the use of local cultivars to create premium products and in the molecular analysis of their prized organoleptic traits [6].

The genetic analysis of grapevine has been hindered by the long generation time (3 years), extensive heterozygosity and phenotypic plasticity. Even berries of the same cultivar may differ markedly in their properties because of environmental factors, from which arises the concept of terroir in viticulture [7]. Detailed characterization of the genome is therefore necessary to separate the genetic and environmental components underlying the phenotype. Grapevine was the first fruit species to be sequenced, but the reference genome is that of a near-homozygous and non-cultivated accession, PN40024 [8]. This resource has facilitated the detailed phylogenetic analysis of specific gene families [9,10], the creation of SNP catalogs which can be used as genetic markers for cultivar differentiation [11] and the development of microarrays for transcriptomic analysis [12,13], but recent deep sequencing experiments have shown that relying on a single reference genome may underestimate the variability among different genotypes. The comparison of Asian and African human genomes with the reference sequence has revealed 5 Mb of novel sequence in each assembly containing population-specific coding regions [14]. Furthermore, the assembly of genomes and transcriptomes from 18 different Arabidopsis thaliana ecotypes led to the identification of 221 genes that are not present in the Col-0 reference [15]. These data indicate that the comparison of polymorphisms in a reference genotype may not represent the full genetic diversity of a species, and this is particularly relevant in grapevine because V. vinifera is more genetically diverse than both Homo sapiens and A. thaliana [2,16]. These challenges could be addressed by *de novo* sequencing and annotating each grapevine cultivar. *De novo* assembly of a complex genome is however hindered by repetitive DNA sequences and low complexity regions. In order to address these problems multiple paired-end and mate-pair libraries with different insert sizes are necessary but require consistent efforts to be produced [17]. Moreover, gene annotation of *de novo* assembled genomes is a time and labor intensive task which includes both the use of gene prediction and annotation methods and a lengthy manual curation.

An alternative to whole-genome sequencing is the direct reconstruction of the transcriptome by *de novo* assembly. The potential of this approach has been demonstrated in animals lacking reference genome sequences such as the coral Acropora millepora [18], the whitefly Bemisia tabaci [19], the butterfly Melitaea cinxia [20], the mosquito Anopheles funestus [21] and the planarian Schmidtea mediterranea [22,23]. Transcript sequences generally lack the repetitive sequences that complicate genome assembly.

*De novo* transcriptome assembly was used to characterize the varietal diversity of V. vinifera cv. Corvina, an indigenous cultivar of the Verona area in north Italy which has recently been subject to comprehensive transcriptomic, proteomic and metabolomic analysis [24-28], including an RNA-Seq based expression profiling of berry development [29]. The direct comparison of potential polymorphisms by projection onto the reference genome indicated that up to one third of the PN40024 proteins could be affected by disruptive mutations, suggesting that a full reconstruction and re-annotation of Corvina genes is required. The *de novo* transcriptome assembly strategy allowed us to identify 19,517 novel splice isoforms among 9463 known genes, and 2321 potentially novel protein-coding genes in the raw PN40024 reads but not in the assembled sequence. We also identified 180 apparently private Corvina genes, 27% of which are modulated during berry development and withering.

## Results

### Sequencing the Corvina transcriptome and the characterization of sequence variation

**Figure 1:**
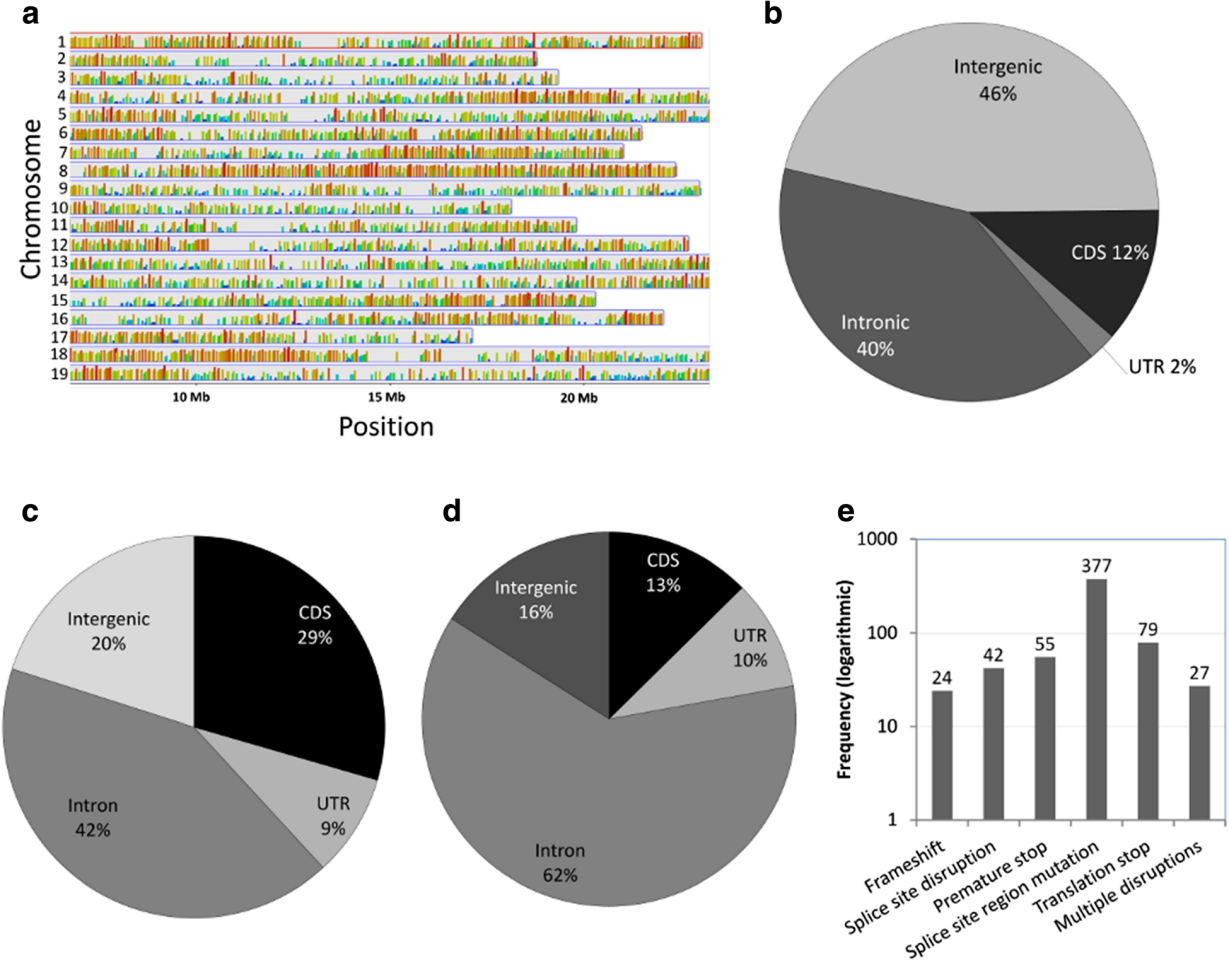
**Genome coverage and sequence variation. a)** Read counts normalized to gene length and log transformed (base=10) respect to the position on the 19 grape chromosomes. **b)** Classification of bases covered (≥3X) by feature type. **c)** Classification of SNPs based on the PN42004 genome annotation. **d)** Classification of indels based on the PN42004 annotation. **e)** Number of genes containing potentially disruptive mutations, plotted in logarithmic scale.

To characterize the V. vinifera cv. Corvina transcriptome, 45 samples were collected from different organs/tissues at several developmental stages (Additional file 1: Table S1). Total RNA from pooled samples was used to generate a single cDNA library with a mean insert size of 310 bp estimated by mapping the reads onto the PN40024 reference genome [8]. Sequencing generated 114,726,580 paired-end reads 100 bp in length, equivalent to 23 Gb of total sequence data. The sequences were quality filtered and the resulting 87,308,996 paired-end reads were aligned to the 450-Mb PN40024 reference genome (12X assembly) with a success rate of 89%. This analysis allowed us to determine the pervasiveness of transcription in terms of the entire gene catalog and to estimate the extent of transcriptional overlap between Corvina and the PN40024 reference genome. We found that 54.7% of the reference genome was covered by at least three reads (Figure 1a) and the proportion did not change significantly by increasing the coverage threshold to 6 or even 10 reads (data not shown). In comparison, at least 63% of the mouse genome [30], 93% of the human genome [31] and 38% of the rice genome is transcribed [32]. However, it should be noted that for human genome only 10 chromosomes were used, accounting for 30% of the entire genome, while for rice genome the coverage was at least 1 read and thus any comparison should be treated with care. The covered bases included approximately 123 Mb of non-annotated sequence, representing 46% of the total and probably reflecting both transcriptional noise and the presence of noncoding RNAs, transposable elements and non-annotated genes in the reference sequence (Figure 1b). The covered bases also included 106 Mb of introns, representing 40% of the total and suggesting extensive differences in alternative splicing between the Corvina cultivar and PN40024 (Figure 1b).

We identified 646,982 polymorphisms between the Corvina and PN40024 sequences, including 137,871 insertion/deletion polymorphisms (indels) and 509,111 single nucleotide polymorphisms (SNPs). Approximately 13% of the indels and 29% of the SNPs were located in regions annotated as coding sequences in the V1 reference annotation (http://genomes.cribi.unipd.it/[33]; Figure 1c,d). We further filtered this dataset with a minimum frequency threshold (≥0.75) of the alternative polymorphism calculated on the total of read pairs aligning on the region This final set contained 67,281 putative mutations, of which 59,064 are SNPs and 8217 indels. Putative mutations were annotated to determine their potential effect on the encoded proteins (Additional file 2: Figure S1). A simple projection of the polymorphisms onto the PN40024 annotation showed that 5808 Corvina proteins were potentially changed by substitutions and 579 proteins were potentially destroyed by frameshifts, premature stop codons, stop-codon mutations and mutations at splice sites (Figure 1e). These data are reminiscent of the situation reported in different A. thaliana ecotypes supporting earlier claims that reference annotations cannot be transferred reliably to any cultivar/accession without the reassembly and re-annotation of the genome and/or the transcriptome [15].

### Reconstruction of the Corvina gene catalog and comparison with reference annotations

The Corvina transcriptome was reconstructed without the reference sequence and annotation by using a two-step strategy involving 87.3 million filtered high-quality paired-end reads. First we generated a preliminary assembly using Velvet, incorporating 77.0 million reads (88.2%) and generating 172,826 contigs. These contigs were then processed with Oases to produce the final set of contigs. Having been assembled from transcriptomic sequence reads, they can be referred to as “putative transcripts”. This analysis produced 140,862 putative transcripts with a minimum length of 200 bp (Table 1), these were clustered and the longest fragment in each cluster was retained, returning 106,670 clusters, each representing a single putative transcript (Table 1). The assembly achieved an average length similar to that of the annotated dataset (1308 vs. 1331 bp) with a slightly higher N50 score (2098 vs. 1755 bp) suggesting that most of the reconstructed putative transcripts were essentially complete.

**Table 1:** General statistics of grape V1 annotations and contig assemblies

	**Grape V1 annotation**	**Velvet assembly**	**Oases deconvolution**	**CdHit Clustering**
**Number of sequences**	29971	172826	140862	106670
**Maximum length**	40713	3541	18312	18312
**Minimum length**	18	200	200	200
**Average**	1331.07	345.57	1392.25	1307.56
**Median**	1126	282	1022.36	917
**N50**	1755	347	2163	2098
**N90**	738	219	678	607

Assembly was performed with Velvet and Oases. Clustering was carried out using CdHit.

**Figure 2:**
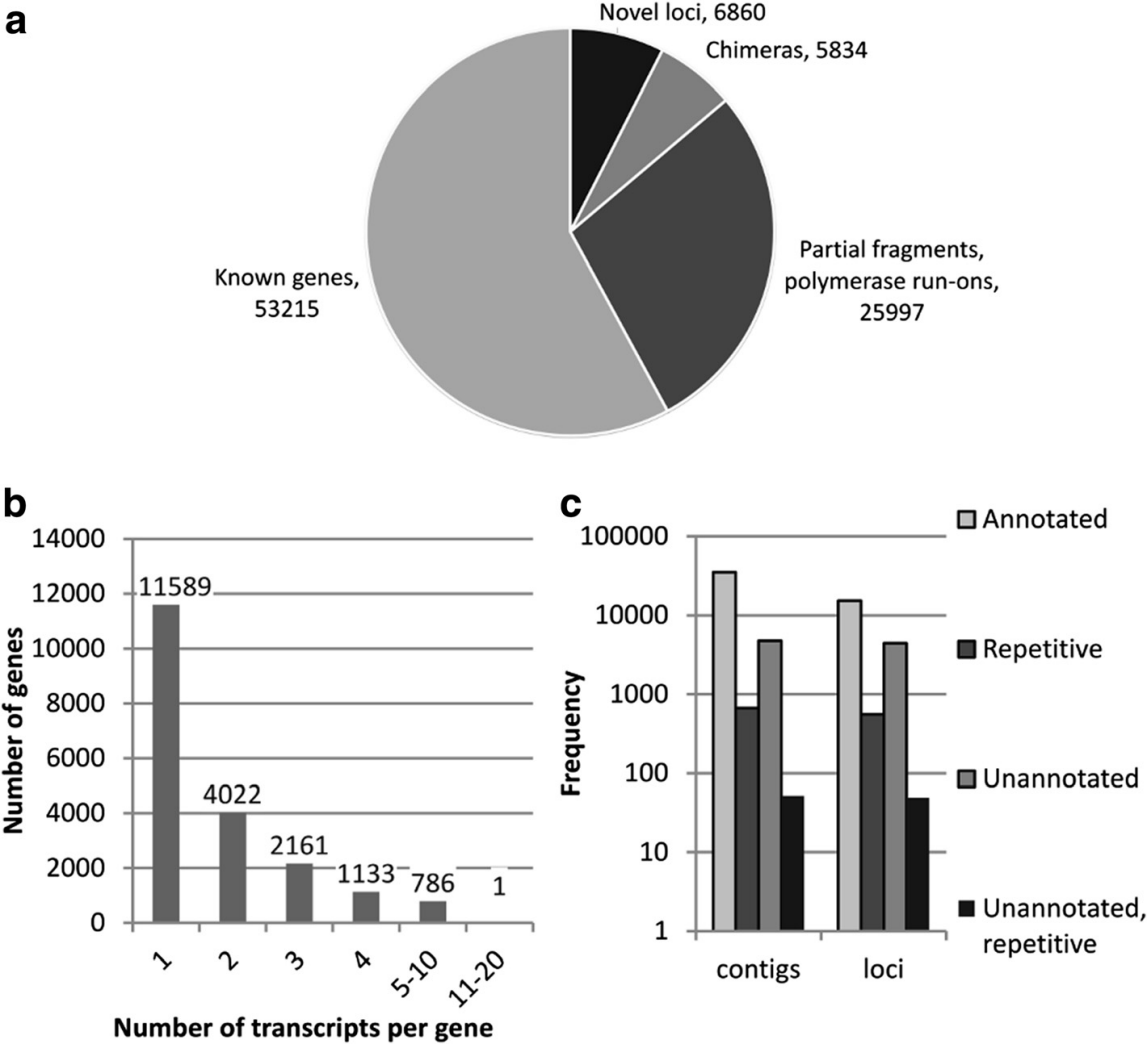
**Contigs classification. a)** Classification of contigs mapping onto the genome based on a comparison with the reference annotation V1 of assembly 12X. **b)** Distribution of the number of contigs per gene after filtering contigs for expression relative to the major isoforms (FMI). **c)** Classification of contigs and respective loci based on genomic region classes.

Our putative transcripts were aligned onto the genome to assign them to distinct genomic loci. In this way, 91,906 putative transcripts were mapped onto the genome with ≥ 90% identity and coverage, including 5834 classified as chimeras. These were excluded from subsequent analysis. Another 25,997 putative transcripts corresponding to single exons, exon fragments or partial introns were removed from the dataset. Of the remaining 60,075putative transcripts, 53,215 were assigned to regions encoding known genes and 6860 were detected in non-annotated regions (Figure 2a). The expression levels of all putative transcripts were calculated and 19,465 putative transcripts were discarded as potential pre-mRNAs because their expression level fell below 5% of the major isoform of the corresponding gene [34] (Figure 2b). This filter increased the percentage of potential protein-coding genes from 83% (no filter) to 88% [35]. We tested higher thresholds (10% and 15%) but this adversely affected the recovery of potential protein-coding genes from the dataset (87% and 86% respectively).

Our final set of putative transcripts were compared with the raw read alignments, revealing a strong reduction in the signal representing intergenic and intronic regions (−96% and −99% respectively), but an increase in the signal representing annotated exons (from 9 to 11.3 Mb, considering only primary alignments). We also compared putative transcript mappings with aligned public EST/cDNA data (http://urgi.versailles.inra.fr/gb2/gbrowse/vitis_12x_pub/), and found that 31,815 putative transcripts overlapped at least one of the 594,733 ESTs, and 10,268 were supported by at least one of the 68,082 available grapevine cDNAs (Table 2).

**Table 2:** Comparison of contig mapping coordinates with grapevine annotations, public ESTs and cDNAs

	**12X assembly**			**8X assembly**
**Contigs classification (12x assembly; V1 annotation)**	**EST**	**cDNA**	**v0 annotation**	**v1 annotation**
Contigs assigned to known genes	29442	9968	31221	23905
Contigs assigned to novel genes	2373	300	935	1635
Contigs assigned to private genes	**NA**	**NA**	**NA**	13

Contigs coordinates were compared with those of public ESTs, cDNAs and V0 annotation of 12x assembly and with v1 annotation of 8x assembly. Number of contigs with at least a 50% overlap with the features of interest is reported for each comparison.

The coordinates of the mapped putative transcripts were then compared with the current V1 annotation (http://genomes.cribi.unipd.it/). The genomic coordinates of the final set of 40,610 putative transcripts corresponded to 17,425 annotated gene loci, 554 known repetitive regions and 4488 putative novel gene loci (Figure 2c; Additional file 3: Table S2; Additional file 2: Figure S2). A similar number of putative novel gene loci (4431) was obtained by a reference alignment and prediction approach starting from the same dataset [33] (data not shown).

By comparing our putative transcripts to the corresponding known genes, we identified 3788 adjacent genes that merged into 1677 putative loci. We compared our 1677 putative loci with the group of 1429 genes that were recently shown to be erroneously split in the V1 annotation [36], finding 346 of the loci in common between the two studies. Based on genome alignments of reconstructed contigs, we compared their structures with known transcripts and were able to identify 5383 *de novo* reconstructed transcripts with an exon-intron structure identical to known PN40024 transcripts. Finally, the analysis allowed us to identify 19,517 novel isoforms representing 9463 genes annotated in the PN40024 reference sequence, 7902 of which generated multiple isoforms (Additional file 2: Figure S2).

**Figure 3:**
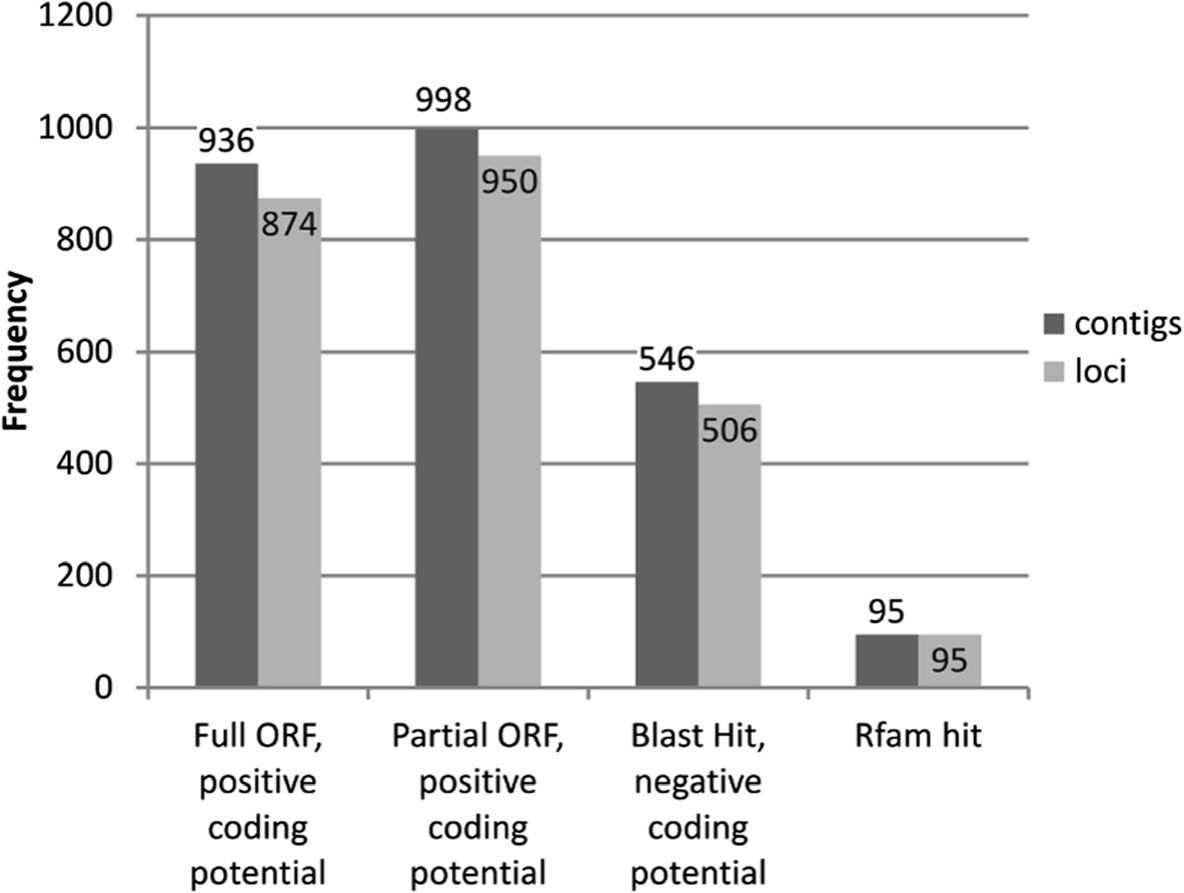
**Classification of contigs mapping in putative novel loci.** Classification of contigs mapping in putative novel loci, based on the coding potential calculated by CPC and on comparison with NCBI nr plant proteins and Rfam databases. Potentially coding contigs were classified as full ORFs begin with a start codon and end with an in-frame stop codon, or as partial ORFs if one of these two features was missing. The following two categories include contigs with a negative coding potential but Blast hit against the NCBI NR protein database or contigs with similarity with sequences in the Rfam RNA database.

**Figure 4:**
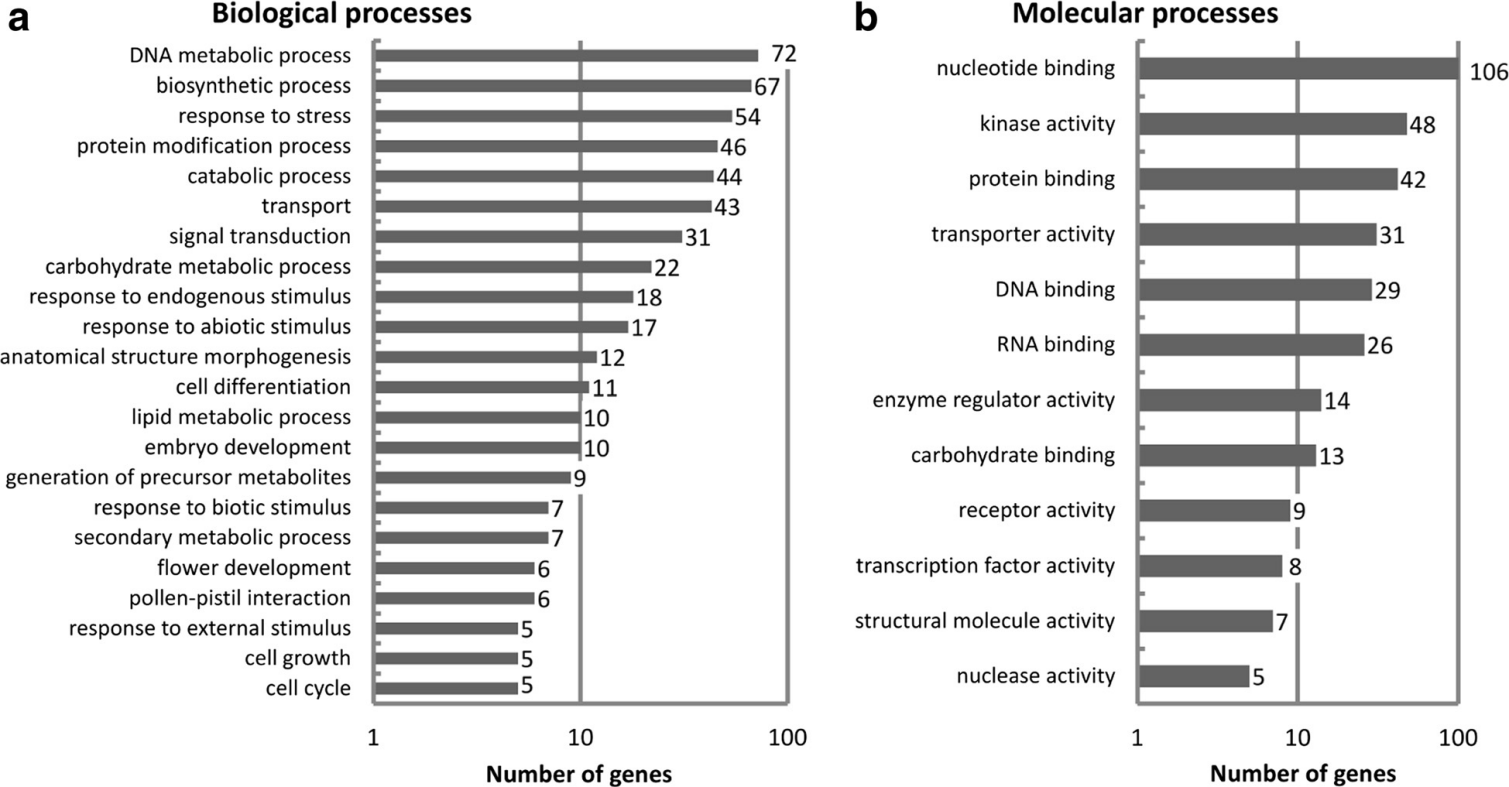
**Gene ontology (GO) classification of novel loci.** Classification based on GO terms of 486 out of 2249 potentially novel protein-coding genes associated to least one GO term (level >1). **a)** Number of assignments to biological process GO onthology terms. **b)** Number of assignments to molecular function GO onthology terms.

Among the 4488 potential novel gene loci, 49 showed significant sequence similarity to transposon or retrotransposon proteins, 1785 were identified using Coding Potential Calculator (CPC) and 464 generated hits when used as BLAST queries against the NCBI non-redundant protein database (E-value ≤ 1x 10^-5^). The remaining 2190 loci were considered to be putative non-coding RNAs (Figure 3). Functional annotations were applied using Gene Ontology (GO) classifications, revealing 426 of 2249 loci that were associated with at least one GO term (GO level >1) (Figure 4). Finally, the putative transcript coordinates of novel genes were compared with V0 (http://www.genoscope.cns.fr/spip/Vitis-vinifera-whole-genome.html) and 8x annotations [8] (Additional file 3: Table S2).

### Identification of Corvina private genes

**Figure 5:**
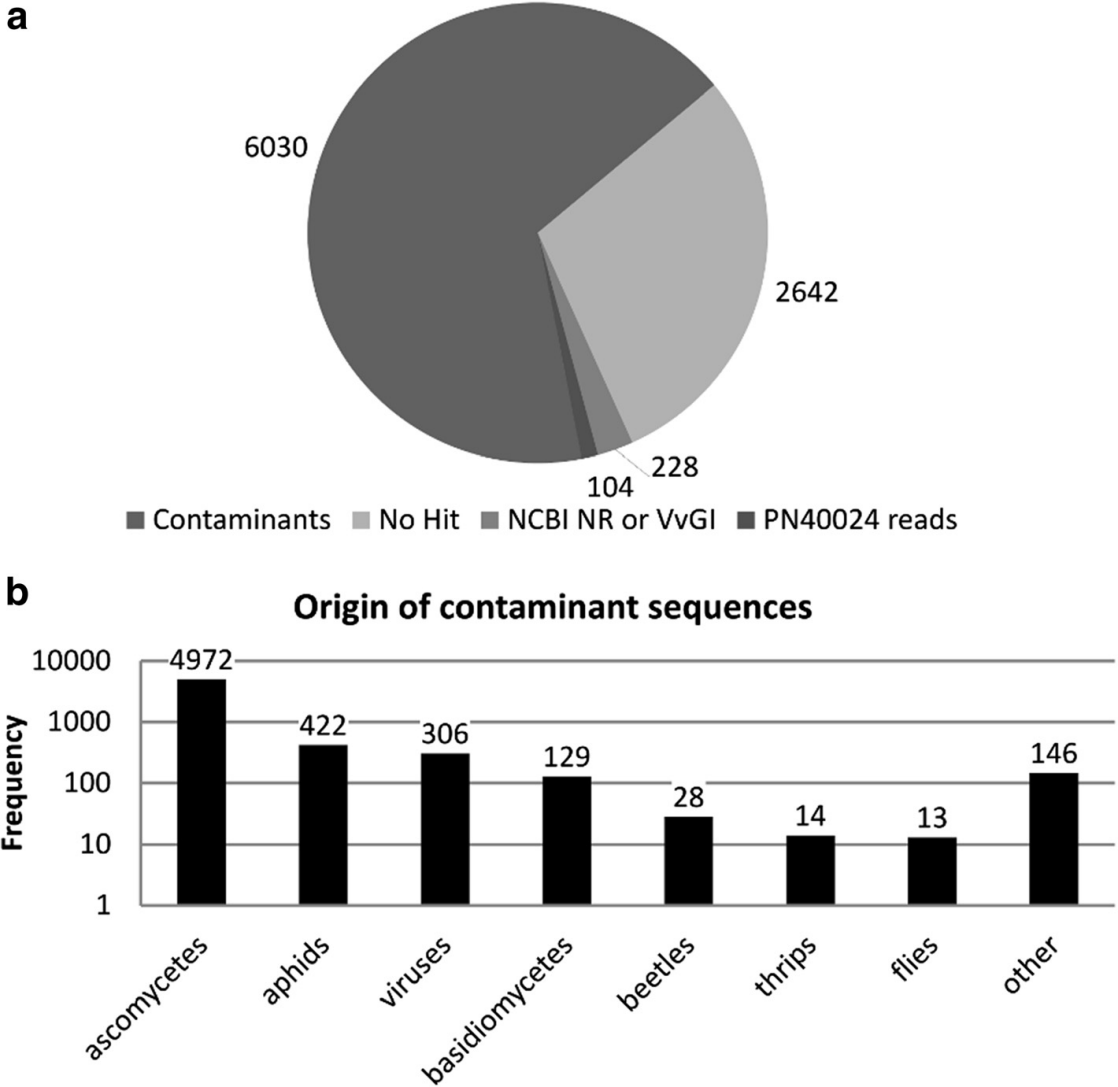
**Classification of contigs not mapping onto the reference genome. a)** Distribution of unmapped contigs based on similarity to sequences in the NCBI non-redundant protein database and nucleic acid sequence databases (VvGI 8.0 and PN40024 raw reads). **b)** Distribution of contaminant sequences across different taxa.

**Figure 6:**
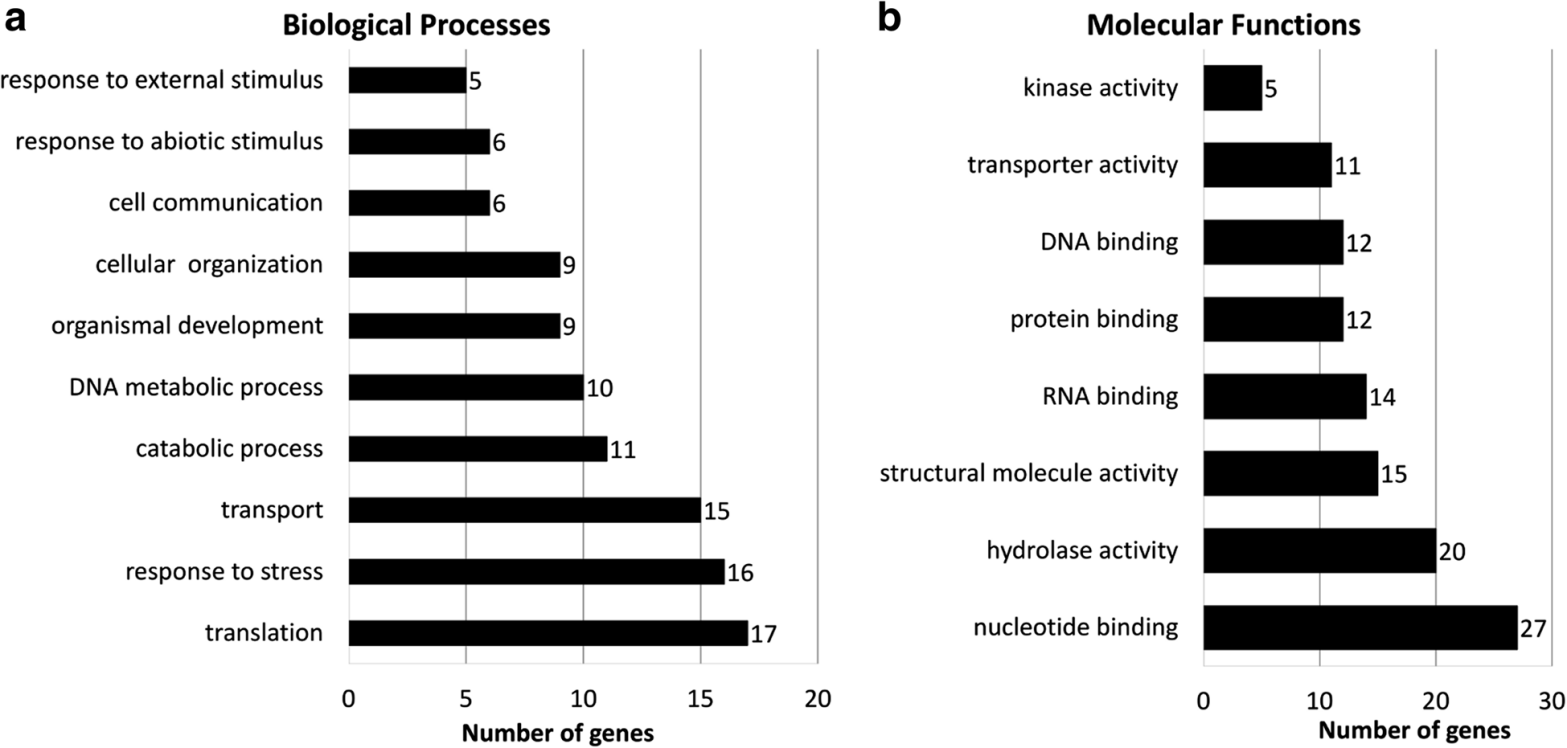
**Gene ontology (GO) classification of private genes.** Assignments to GO terms of 100 out of 180 contig clusters corresponding to putative private genes. **a)** Number of assignments to biological process onthology terms. **b)** Number of assignments to molecular function onthology terms.

Of the 9004 identified putative transcripts that could not be mapped onto the PN40024 genome (Figure 5a), 6030 were discarded as contaminants because most (82%) appeared to be fungal in origin (Figure 5b). We found that 332 of the remaining 2974 putative transcripts matched expressed grapevine sequences represented in the VvGI database v8.0 or other plant proteins, and these were considered as novel grapevine transcripts potentially restricted to V. vinifera cv. Corvina (Corvina private genes). To avoid false positives reflecting gaps in the PN40024 gene space, we compared the 332 potential Corvina private genes with PN40024 raw sequence reads and found matches for another 104 sequences, 72 of which were potential coding regions that might represent previously undiscovered genes in the PN40024 genome. The remaining 228 putative transcripts appeared to be Corvina-specific, and formed 180 clusters based on similarity to sequences present in the VvGI database v8.0 and other plant proteins. These 180 clusters correspond to 180 putative private genes. CPC indicated that 143 of the clusters had a high coding potential [35] and GO classifications indicated preliminary functional classifications for 100 of the sequences (GO level > 1) (Figure 6).

### Dynamic gene expression during Corvina berry development and withering

We assessed the biological significance of the novel genes and Corvina private genes by analyzing berry samples at two developmental stages and one withering stage. Three biological replicates were collected at each stage and were processed to generate indexed RNA-seq libraries, which were sequenced using an Illumina HiSeq 1000. We obtained 147 million 50-bp paired-end reads (14.7 Gb), comprising 46.5 million reads representing the post fruit-set (PFS) stage, 34.7 million reads from the pre-ripening (PR) stage and 65.9 million reads from the post-harvest withering (PHWII) stage (Additional file 4: Table S3).

Gene expression levels at all three stages were quantified using the PN40024 genome and the Corvina private genes as reference sequences. The abundance of each transcript was expressed as fragments per kilobase of exon model per million mapped reads (FPKM) as implemented in Cufflinks [34]. A gene was considered to be expressed if the FPKM 95% confidence interval lower boundary was greater than zero and if the FPKM value was higher than 0.001. We detected 23,538 expressed genes in at least one of the three samples, including 1226 of the 2353 novel genes and 108 of the 180 Corvina private genes. This represented 72% of the grapevine transcriptome (including novel and private genes). Although more genes were detected in the berry samples than in the reconstructed pool, 5264 genes expressed in the pooled samples were not represented in any of the three berry samples.

**Figure 7:**
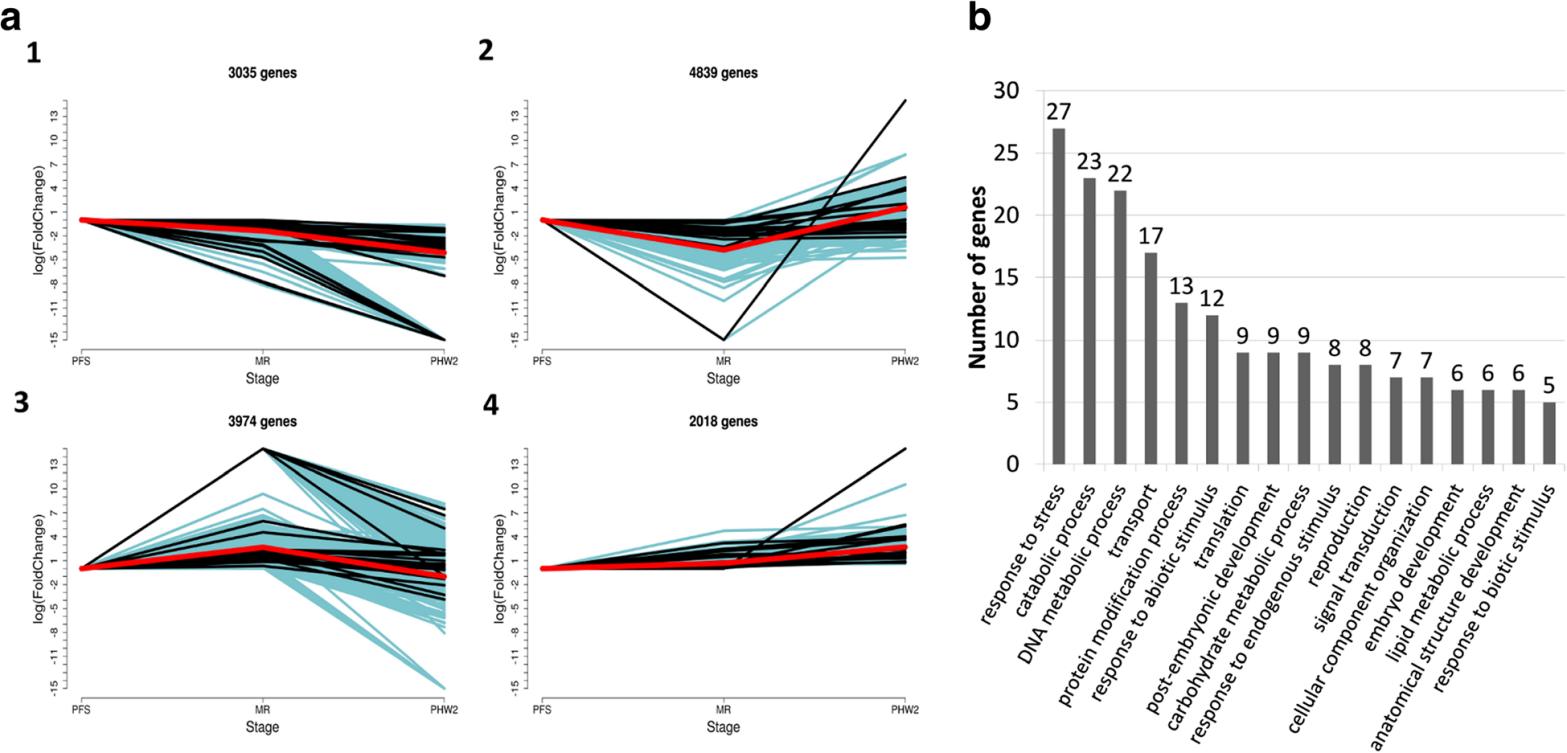
**Expression profiles of 13,886 genes differentially expressed during berry development and withering. a)** The differentially expressed genes were divided into four groups according to the expression profile: 1) repressed genes; 2) transiently repressed genes; 3) transiently induced genes; 4) induced genes. Black: novel gene loci in un-annotated regions of the genome; blue: putative private genes; red: average profile of the expression group. **b)** Number of assignments to GO Slim plant terms for the novel or private genes differentially expressed among at least two stages.

Raw counts of uniquely-mapped reads were estimated and normalized [37]. We identified 13,866 loci that were modulated in at least one of the samples (FDR ≤ 0.05% and |log2 fold change| ≥ 1 at one or more time points) (Figure 7). At each time point, we detected both unique and overlapping sets of differentially-expressed genes, e.g. 5716 genes were differentially expressed in all three stages analyzed and 6493 were differentially expressed specifically during withering.

Interestingly, 50 of the 180 Corvina private genes were differentially expressed and 15 (8.3%) were induced specifically during withering. Similarly, 524 of the 2353 novel loci were differentially expressed in at least one sample. Genes were grouped according to their expression profiles by transforming the expression data into moderate fold change estimates using a variance stabilizing transformation [37]. Differentially-expressed genes were thus grouped into four clusters according to their expression profiles, comprising those repressed at PR and PHWII or exclusively at PHWII but not PFS (cluster 1), those transiently repressed at PR (cluster 2), those transiently induced at PR (cluster 3) and those induced at the PR and PHWII stages or at the PHWII stage alone (cluster 4) (Figure 7a).

To gain insight into the functions of the modulated genes in each cluster, each group was enriched with GO terms (FDR <5%) associated with 10,842 of the 13,866 differentially-expressed genes (Additional file 5: Table S4). Looking specifically at genes induced during the PHWII phase (24.79% of the total) given that withering is peculiar to wines produced from this cultivar, the statistics show an enrichment for genes involved in stress responses such as programmed cell death and in the synthesis of flavonoids, as previously described [25].

## Discussion

Sequence diversity is usually described in comparison to a reference genome [38,39]. Given the high degree of genetic diversity among plant cultivars, this approach might fail to recognize highly polymorphic regions and will not detect the presence or absence of genes residing in private (cultivar-specific) regions of the genome [15]. Whole-genome sequencing and re-annotation is therefore recommended for each variety, but in predominantly heterozygous species such as grapevine the sequence diversity would make contig assembly a daunting and resource-intensive task [8].

When a reference genome is available, genes and transcript isoforms are built *de novo* by mapping RNA-seq reads, but this does not solve the problem of hypervariable sequences and private genes [34,40]. However, the *de novo* assembly strategy does not depend on the genome and has been applied successfully to reconstruct the transcriptomes of non-model species lacking reference genomes.

We have demonstrated the feasibility cDNA sequencing by RNA-seq for the analysis of varietal diversity between a local grapevine cultivar (Corvina) and the PN40024 reference genome without genomic data. The availability of a reference genome allows the reconstruction procedure to be validated and highlights the diversity between the two genomes.

### Improved annotation of the reference genome

The latest grapevine genome annotation (v1 produced by CRIBI; http://genomes.cribi.unipd.it/) comprises 29,971 genes identified by a combination of ab initio prediction and cDNA mapping. By comparing this annotation to the transcripts we identified, we found our method had detected 51% of the annotated genes, the remainder probably representing tissue/condition-specific transcripts that were not present in our pooled samples. The genes overlapping our sample and the v1 annotation have a higher expression level than the v1-specific genes (mean = 35.67 vs 14.31 FPKM, median= 13.03 vs 1 FPKM). These data indicate that many of the v1 annotations undetected using our method were missed because of the paucity of sequencing reads generated from the corresponding loci. A large number (2249) of potential protein-coding genes were detected in the non-annotated parts of the genome. A recent comparison of the 8x, 12x v0 and 12x v1 annotations showed that 6089 genes present in either the 8x or 12x v0 assemblies were not present in the v1 annotation [36]. Interestingly, 1171 of our 2353 potential protein-coding genes (72 of which are only present in raw reads) were represented in the 8x or 12x v0 annotations. Current annotation is therefore incomplete and insufficient to describe the full gene space of a cultivar other than the reference Pinot Noir clone. Our method provided experimental support for 72 protein-coding genes missing from the final assembly because they were excluded from the 12x consensus, and for 2249 additional genes that appear to have been missed in the v1 annotation. Novel genes excluded from the v1 annotation appear to have meaningful biological roles, including those modulated during berry ripening and/or withering e.g. eight disease-resistance genes (Novel_1755, Novel_2241, Novel_0853, Novel_2382, Novel_2375, Novel_1428, Novel_2207, Novel_1998), two stress-inducible genes (Novel_4520 and Novel_4511), a heat shock protein 70 gene (Novel_4478) and a senescence-associated gene (Novel_1324). The expression of the disease-resistance genes generally declined during berry development and withering (clusters 1 and 2) suggesting their role is to protect the berry from pathogens and pests during early development. In contrast, the stress-inducible genes and heat shock protein gene were induced during ripening and withering, supporting a protective role against abiotic stress during the accumulation of sugars and secondary metabolites as previously reported [25,41,42]. The RNA-seq data therefore provide a comprehensive insight into the biologically-relevant landscape of gene expression during berry development and ripening.

Our method not only offers a way to annotate previously uncharacterized genes but also improves the annotation of known genes by helping to define their boundaries more robustly and to identify splice variants. Our data indicate that up to 11% of the genes in the v1 annotation are split incorrectly, similar to the error rate in other annotated plant genomes [43]. A previous in silico analysis identified 1429 instances of erroneously split genes in the v1 annotation [36]. We also detected 462 of these genes and our analysis suggested that 75% of them were split incorrectly in the v1 annotation. Furthermore, our data resulted in the 3′ and 5′ extension of nearly 90% of the genes we detected compared to the boundaries in the v1 annotation, indicating that the untranslated regions were longer than previously reported, using in silico prediction methods [44]. Our approach may therefore provide a useful complement to ab initio gene prediction methods to establish gene boundaries and define UTRs. Finally, our *de novo* transcriptome assembly method detected an average of 1.75 transcripts per locus, in line with previous reports using a reference-guided assembly of grapevine transcripts (1.25 transcripts per locus [45]). Although beyond the scope of our investigation, the *de novo* reconstruction indicated alternative splice variants for 9463 loci, providing a much more exhaustive description of the grapevine transcriptome compared to in silico predictions. The number of studies which try to describe alternative splicing events in plants are still scarce, however many recent studies point to an extensive diffusion of the phenomenon and to its important role in modulating gene expression and stress response ( [46-48]). Our results indicate that the transcriptional landscape in Vitis is more complex than previously thought and therefore warrants further investigation.

### Expression of Corvina private genes during berry development

Recent data from the deep sequencing of human individuals and Arabidopsis ecotypes revealed portions of genome that are not shared among all genotypes and the reference genome [14,15]. Interestingly, the novel genomic sequences included a set of protein-coding genes (private genes) potentially contributing to the intra-species variability. Similarly, we detected 180 putative protein-coding genes with a high coding potential or matches to plant ESTs that represent potential Corvina private genes.

We identified 146 private genes expressed in at least one berry-sampling phase, 50 of which were differentially expressed between samples, and these could represent a group of genes that directly contribute to the specific characteristics of the Corvina berry. Some of these private genes could have been selected by ancient breeders looking for particular berry quality traits, such as the ability to withstand the lengthy drying phase (rasinate) required to make passito wines (straw wines) such as Amarone and Recioto. For example, we identified a heat shock protein gene (Private_087) and a stress-inducible gene (Private_101) induced during ripening, consistent with the ability of Corvina berries to undergo dehydration for up to 100 days [26,27]. Furthermore, we detected the induction of genes involved in translation and protein metabolism during withering, including three ribosomal proteins (Private_068, Private_108 and Private_116), three elongation factors (Private_166, Private_164 and Private_152), ubiquitin (Private_122), a 5-methyltetrahydropteroyltriglutamate-homocysteine methyltransferase (Private_094) and a DNA-binding protein (Private_171). This supports cDNA-AFLP data indicating the induction of genes with similar functions during withering [25].

Thirty-three of the Corvina private genes matched homologs in other grape varieties but not the reference genome. This is expected because the dispensable part of the genome may be partly shared among different cultivars and only a few genes may be truly unique to a particular accession [15]. For example, we found two Flowering Locus T (FT) genes (Private_100 and Private_113) the first corresponding to the previously-described VvFT gene found in the cultivars Cabernet Sauvignon [49] and Tempranillo [50]. At least six members of the FT/TFL1 gene family were identified in the Tempranillo genome, including VvFT which appears to be the ortholog of Arabidopsis FT and therefore induces precocious flowering when expressed in Arabidopsis, consistent with reported expression patterns associated with seasonal floral induction in latent buds and with the development of inflorescences, flowers and fruits [50]. There is no evidence for the presence of classical floral regulatory pathways in grapevine, and the expression profile of VvFT suggests that it only partially corresponds to the florigen role of Arabidopsis FT. We also observed the expression of VvFT during berry formation, suggesting an additional and uncharacterized role of this gene during early berry formation.

## Conclusions

We were able to reconstruct a substantial part of the grapevine transcriptome (51% of known genes), improve the annotations of known genes by defining their boundaries and splice variants, add 2353 apparently novel genes representing non-annotated or unassembled regions of the reference genome, and also add 180 potentially Corvina-specific private genes that are not present in the reference sequence. Our results are consistent with data from other plant species showing that different genotypes share a common majority of genes but also possess smaller sets of private genes that are likely to be dispensable, that contribute to intra-specific variation and that produce unique, variety-dependent characteristics [15,51]. Given the substantial divergence among registered ecotypes and cultivars [52,53], we argue that in plant biology a *de novo* transcriptome assembly approach should not be limited to species lacking reference genome (e.g. [21,54,55]) because it can improve the annotation of diverse cultivars and identify cultivar-specific private genes without embarking on a labor-intensive reconstructing of the entire genome.

## Methods

### Sample collection

To cover most of the grapevine transcriptome, we created a pool of RNA samples representing different organs and developmental stages of V. vinifera cv Corvina (clone 48). We selected 45 of the 54 samples described by Fasoli et al. [13] and combined 1 μg of total RNA from each sample (Additional file 1: Table S1). Berries were collected from a vineyard in Verona (Italy) at three time points: post-fruit set (PFS), mid-ripening (MR) and mid-withering approximately 2 months post-harvest (PHW II). At the PFS stage (35 days after flowering (DAF); E-L 32), the berries were >7 mm in diameter and touching, whereas at the MR stage (84 DAF; E-L 36) they had reached their final size and the sugar content was 15.5° Brix. At the PHW II stage, the berry weight was 69.7% of the weight at harvest and the sugar content was 25.9° Brix (ripe values). The sugar content (mean Brix degree value) was recorded at each time point using a PR-32 bench refractometer (Atago Co., Ltd, Tokyo, Japan).

### RNA extraction

Total RNA was isolated from ~200 mg of the ground berry pericarp using the Spectrum™ Plant Total RNA kit (Sigma-Aldrich, St. Louis, MO) following the manufacturer’s protocol. RNA quality and quantity were determined using a Nanodrop 2000 spectrophotometer (Thermo Scientific, Wilmington, DE) and a Bioanalyzer Chip RNA 7500 series II (Agilent, Santa Clara, CA).

### Library preparation

Total RNA samples were assessed for quality using an RNA 6000 Nano Kit (Agilent, Wokingham, UK) and 2.5-μg aliquots were used to isolate poly(A) mRNA for the preparation of a non-directional Illumina RNA-seq library using the TruSeq RNA Sample Prep Kit v2 (Illumina Inc., San Diego, CA, USA). The quality of the library was checked with a High Sensitivity DNA Kit (Agilent, Wokingham, UK). Libraries were sequenced with an Illumina HiSeq 1000 sequencer (Illumina Inc., San Diego, CA, USA) and 100-bp paired-end sequences were generated.

### Pre-processing of reads

Low-quality reads (> 50 bases with quality < 7 or > 10% undetermined bases) and putative PCR duplicate reads were removed and Illumina TruSeq adapter sequences were clipped. Low-quality bases at read ends were trimmed (minimum quality 16, minimum read length 50 bp) with cutadapt (http://code.google.com/p/cutadapt/).

### Mapping and polymorphism detection

The mean read insert size and standard deviation were estimated using a BWA alignment [56] with default parameters of a random sample of one million reads against the 12x PN42004 grapevine genome. The reads were then aligned using TopHat v1.3.0 [57], giving as parameters the derived mean read spacer size (110 bp) and its standard deviation (189 bp). Polymorphisms were called using FreeBayes [58] with default parameters and further filtered for read depth ≥ 5 and a polymorphism call quality ≥ 80 with vcftools [59]. A custom script (vcf_filter.py) was used to select putative mutations based on a frequency of the alternative alleles (≥ 0.75) calculated on the total number of read pairs aligned on the region. The putative mutations were annotated using the Variant Effect Predictor on EnsEMBL version 64 [60].

### *De novo* assembly

*De novo* assembly was carried out using the Velvet/Oases package, using a k-mer value of 41, a minimum contig length of 200, an insert length of 310 and a standard deviation of 189 [61]. Multiple k-mers would potentially have given a better sensitivity, especially against low expressed genes. However, the step of k-merge introduces uncertainties (Ns) in the sequences which we preferred to avoid when mapping reads against the contigs to evaluate expression levels. For this reason, a more “conservative” approach was adopted, using just one k-mer optimized by running different assemblies (from 21 to 51). We chose 41 as a tradeoff value, as using higher k-mers improved the N50 and average length by a minimal percentage (<5% passing from 41 to 47; data not shown) while avoiding a loss in sensitivity. As Oases does not cluster assembled contigs if used with only one k-mer, we used CdHit to cluster the Oases contigs with identity >90% and a coverage of 100% [62].

### Sequence mapping

Contigs were aligned against the PN40024 genome [8] using GMAP under the following parameters: -B 4 -t 6 -x 30 -f 2 -t 6 [63]. Sequence alignments with BLAST were carried out using a threshold E-value of 10^-5^ and a minimum alignment coverage of 20% of the query sequence.

### Comparison of annotations

The Tuxedo suite programs Cufflinks 1.4.1 and Cuffcompare (default parameters) [34] were used to annotate contigs against the current V1 grapevine annotation (http://genomes.cribi.unipd.it/) [33]. Comparisons with the 8x V0 annotation (http://www.genoscope.cns.fr/spip/Vitis-vinifera-whole-genome.html) [8] and cDNAs, ESTs and UniProt sequences were carried out with the intersectBed program from the BedTools suite [64].

### Coding potential estimation

The coding potential of each contig was estimated using CPC [35] against the Uniref90 protein database (2012-01-04) [65], with default parameters.

### Differential gene expression analysis

Reads were aligned against the PN40024 genome and the putative new grape transcripts, using TopHat v1.4 [57]. We calculated the FPKM (fragments per kilobase of exon per million fragments mapped) expression values of known genes, new genomic loci, and sequences outside the reference genome using Cufflinks v1.2 with default parameters [34,40]. We used the R package DESeq and its included script [37] to obtain raw read counts from the alignments and to identify differentially-expressed genes (parameters: FDR ≤ 5%, lfc ≥ 1).

### Data access

All next-generation sequencing data are available in the Sequence Reads Archive (SRA) with accession number SRA055265. Contigs assembled from Illumina reads have been submitted to NCBI TSA, under the accession codes KA133930 – KA174709.

## Competing interests

The authors declare no competing interest.

## Authors’ contributions

LV and AF performed the *de novo* assembly, all subsequent computational analyses and the writing of the manuscript. AM helped perform the computational analyses. DB, SZ, MP and GBT assessed the functional role of novel and private genes, analyzed the gene expression patterns in the berry and drafted the manuscript. MF and SZ extracted RNA from all samples. GB, PT, SDS and EDZ performed sequencing library preparation and sequencing itself. GZ assisted in drafting the manuscript. MD designed the study and wrote the manuscript. All authors have read and approved the manuscript for publication.

## Supplementary Material

Additional file 1 **Table S1.** Lists all the tissues and developmental stages from which the pool was constructed. Click here for file

Additional file 2 Includes a first figure describing the mutations detected in Corvina and a second figure describing the distribution of the isoforms on the genome. Click here for file

Additional file 3 **Table S2.** Contains information regarding the novel and private genes detected by the analysis, their annotation and expression values in the three berry developmental stages. Click here for file

Additional file 4 **Table S3.** Enumerates the number of fragments obtained for each berry condition analyzed. Click here for file

Additional file 5 **Table S4.** Contains the GO enrichment analysis for the 4 expression clusters and the genes induced specifically at the PHWII stage. Click here for file
