# Supplementary material for: De novo transcriptome characterization of Vitis vinifera cv. Corvina unveils varietal diversity
Source: BMC Genomics. 2013 Jan 18;14:41. doi: 10.1186/1471-2164-14-41 (PMC3556335; doi:10.1186/1471-2164-14-41)
Supplement: Additional file 2 — Includes a first figure describing the mutations detected in Corvina and a second figure describing the distribution of the isoforms on the genome. [file 1471-2164-14-41-S2.pdf]

Fig. S1

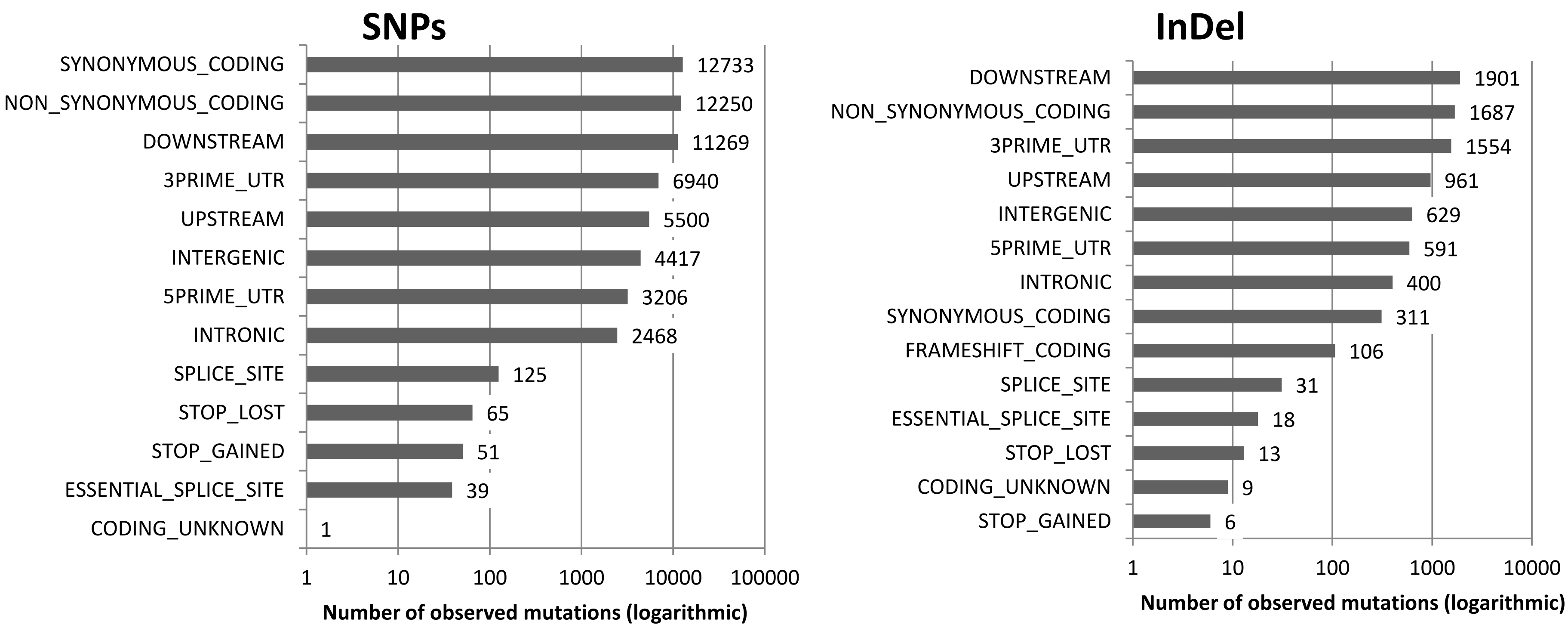

Polymorphisms annotation. Putative mutations detected by comparison of RNA-Seq reads were annotated in order to classify their potential effect on the encoded proteins based on PN40024 reference annotation.

Fig. S2

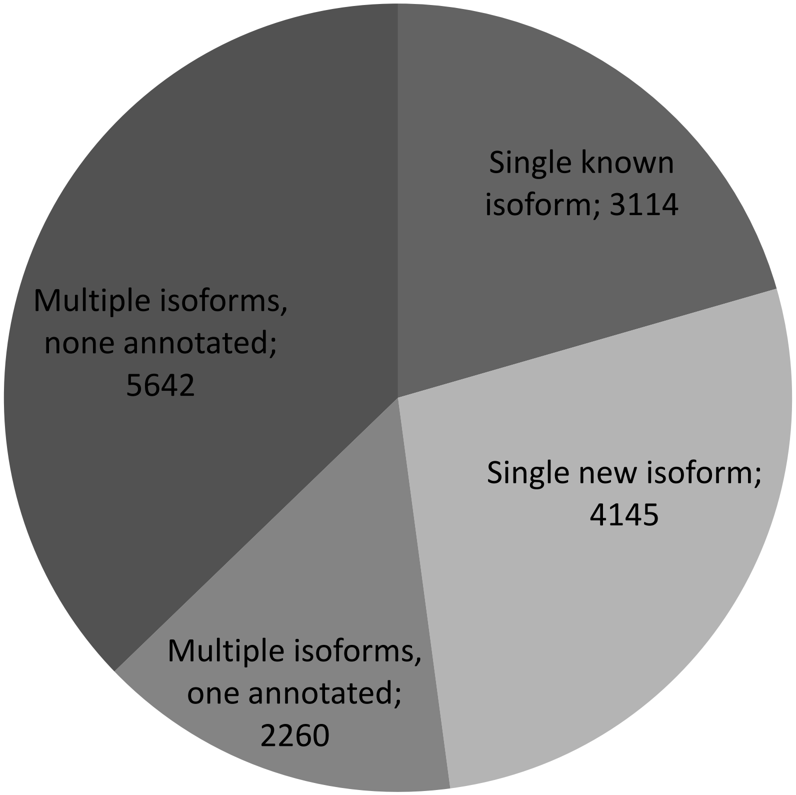

Distribution of isoforms across known genes. Most of them (12,047) have at least one new isoform and 7,902have multiple transcript isoforms.
